# Fabrication and Adsorption Behavior of Magnesium Silicate Hydrate Nanoparticles towards Methylene Blue

**DOI:** 10.3390/nano8050271

**Published:** 2018-04-24

**Authors:** Renyao Huang, Li He, Tao Zhang, Dianqing Li, Pinggui Tang, Yingying Zhao, Yongjun Feng

**Affiliations:** 1 State Key Laboratory of Chemical Resource Engineering, Beijing Engineering Center for Hierarchical Catalysts, Beijing University of Chemical Technology, Beijing 100029, China; huangrenyao203@163.com (R.H.); 13260178198@163.com (L.H.); lidq@mail.buct.edu.cn (D.L.); tangpg@mail.buct.edu.cn (P.T.); 2 Beijing Center for Physical & Chemical Analysis, Beijing 100089, China; zhtao08@163.com

**Keywords:** magnesium silicate, adsorption behavior, methylene blue, surface charge

## Abstract

Magnesium silicate as a high-performance adsorption material has attracted increasing attention for the removal of organic dye pollution. Here, we prepared a series of magnesium silicate hydrates (MSH) in a hydrothermal route, and carefully investigated the corresponding adsorption behavior towards methylene blue (MB) as well as the effect of surface charge on adsorption capacity. The results show that surface charge plays a key role in the adsorption performance of MSH for MB, a negative surface charge density follows the increase of Si/Mg feeding ratio from 1.00 to 1.75, and furthermore the higher negative charge favors the improvement of the adsorption capacity. Among four investigated samples (MSH = 1.00, 1.25, 1.50, and 1.75), MSH-1.75 has the highest negative surface charge and shows the largest adsorption capacity for MB. For example, the equilibrium adsorption quantity is 307 mg·g^−1^ for MSH-1.75, which is 35% higher than that of 227 mg·g^−1^ for MSH-1.00. Besides, for MSH-1.75, the as-prepared sample with negative charge exhibits ca. 36% higher adsorption quantity compared to the sample at the zero point of charge (pH_ZPC_). Furthermore, magnesium silicate hydrate material with Si/Mg feeding ratio = 1.75 demonstrates the promising removal efficiency of beyond 98% for methylene blue in 10 min, and the maximum adsorption capacity of 374 mg·g^−1^ calculated from the Langmuir isotherm model.

## 1. Introduction

Various synthetic dyes have been developed and have played an important role in modern life. During the process of production and application, however, ca. 10% of synthetic dyes are inevitably released to the environment as the main components in effluent wastewaters [1], which have seriously negative influences on the chemical oxygen demand, biological oxygen demand, pH value and translucency to an unacceptable level, and furthermore are threatening the environment and human health [2]. 

The trend in water and wastewater treatment is continuously moving towards more sustainable materials and technologies [3]. Recently, magnesium silicate as a high-performance and sustainability adsorption material has attracted increasing interest to remove heavy metal ions and various kinds of dyes [4,5]. However, most special attentions has been paid to the enhancement of adsorption capacity by increasing surface area and optimizing pore size, such as mesoporous [6], hierarchical [7], nanosized [8], sandwich-like [9], nanotubes [10], and yolk-shell magnesium silicate [11]. Besides the pore structure, the surface properties also play crucial roles in enhancing the adsorption capacity [12,13,14,15,16]. For example, Ferrero [17] considered that the adsorption mechanism of magnesium silicate was concerned with both of the electrostatic attraction and the ion-exchange for methylene blue. To date, few reports have been published on the detailed relationship between surface charge and adsorption behavior on magnesium silicate material.

In this work, we fabricated a series of magnesium silicate hydrate (MSH) adsorbents with different surface charge density by adjusting the Si/Mg feeding ratio in a hydrothermal route, and carefully investigated the corresponding adsorption behavior for methylene blue as well as the relationship between surface charge density and adsorption capacity.

## 2. Experimental

### 2.1. Materials

The magnesium nitrate hexahydrate (Mg(NO_3_)_2_·6H_2_O), sodium silicate nonahydrate (Na_2_SiO_3_·9H_2_O), hydrochloric acid (HCl, 36.5 wt %), sodium chloride (NaCl) and methylene blue (MB) were all analytical grade and directly used as received from ALADDIN (Shanghai, China). The water used in this work was deionized water.

### 2.2. Preparation of Magnesium Silicate Hydrate

Four magnesium silicate samples with different surface charge densities were individually prepared in a hydrothermal route by adjusting the Si/Mg feeding ratio. As for the sample with Si/Mg ratio = 1.75, typically, 10.3 g (40 mmol) Mg(NO_3_)_2_·6H_2_O and 17.1 g (70 mmol) Na_2_SiO_3_·9H_2_O were separately dissolved in 200 mL of deionized water at 25 °C to produce 0.2 mol·L^−1^ Mg(NO_3_)_2_ solution and 0.35 mol·L^−1^ Na_2_SiO_3_ solution. Both of two solutions were mixed under vigorous stirring for 3 min. Subsequently, the resulting white slurry was transferred into an autoclave (1 L) and then kept at 190 °C for 12 h at the stirring speed of 300 rpm. Finally, the sample was collected after being washed by water, centrifuged, and dried at 60 °C for 12 h. Another three samples were synthesized with the similar procedure and same amount of Mg(NO_3_)_2_·6H_2_O but different Si/Mg feeding ratios. Here, four samples were individually obtained and named as: MSH-1.00 for Si/Mg ratio = 1.00, MSH-1.25 for Si/Mg ratio = 1.25, MSH-1.50 for Si/Mg ratio = 1.50, and MSH-1.75 for Si/Mg ratio = 1.75.

### 2.3. Characterization

Crystal structure was determined by X-ray Diffraction (XRD) on a Rigaku UItima III X-ray powder diffractometer (Cu Kα radiation, λ = 0.15406 nm) in the range of 3°~70°/2θ with 10° min^−1^ (Tokyo, Japan). Particle size and size distribution were examined by Malvern Mastersizer 2000-Hydro 2000MU particle size analyzer (Malvern, UK) by dispersing the powder in water. Elemental analysis was performed using a Shimadzu ICPS-75000 (Kyoto, Japan) inductively coupled plasma atomic emission spectrometer (ICP-AES). Morphologies were recorded with a Hitachi S-4700 scanning electron microscope (SEM) (Eindhoven, The Netherlands) at 30 kV and a JEOL JEM-2100F transmission electron microscope (TEM) (Tokyo, Japan). Elemental distribution was tested by a EDAX-GENESIS 60 energy-dispersive X-ray spectroscopy (EDX) (Mahwah, NJ, USA) coupled with SEM. Specific surface area and pore property were analyzed by low-temperature N_2_ adsorption-desorption method on Micromeritics ASAP 2390 (Norcross, GA, USA) volumetric adsorption analyzer. Considering the wide range of pore sizes from 0.5~200 nm, the density functional theory (DFT) was used to calculate pore size distributions from adsorption isotherms based on given intermolecular fluid-fluid and fluid-solid potentials [18]. The pH value was measured by Mettler Toledo FE20 pH meter (Zurich, Switzerland) with LE438 combination electrodes with a resolution of 0.01. Fourier transform infrared spectroscopy (FT-IR) spectra were collected on Bruker Vector 22 infrared spectrophotometer (Karlsruhe, Germany) in the wavenumber range of 400~4000 cm^−1^. 

### 2.4. Surface Charge Studies

The surface charge of the samples was estimated according to the method of potentiometric titration [19]. To further investigate the quantitative change of adsorption capacity caused by surface charge, 32.5 mg of MSH-1.75 was dispersed in a 100 mL flask containing 40 mL deionized water with a preset pH value adjusted by dilute HCl and the same ionic strength by adding NaCl, respectively. Subsequently, nine flasks per pH value were sealed using plastic wrap, put in the thermostatic shaker, and shaken for 12 h at 25 °C and 160 rpm, and the final pH values of the suspension were recorded. Then 10 mL, 1000 mg·L^−1^ MB solution was individually added into these flasks to produce a suspension containing 200 mg·L^−1^ MB. These flasks were sealed using plastic wrap, put in the thermostatic shaker, and shaken for 0~120 min at 25 °C (160 rpm). After a certain time, the suspension was separated by centrifugation (3700 rpm, 5 min), and the rest concentration of MB after adsorption was estimated by a UV/Vis spectrophotometer at λ = 664 nm [20]. The adsorption quantity *q_t_* of the sample at a certain time *t* was calculated using the following equation [21]:

(1) $$q_{t}=\frac{(C_{0}-C_{t})\times V}{m}$$

where *q_t_* (mg·g^−1^) is the adsorption quantity of the sample for MB at the contact time *t* (min), *C*_0_ (mg·L^−1^) is the initial concentrations of MB, *C_t_* (mg·L^−1^) is the rest concentration of MB at the contact time *t* (min), *V* (L) and *m* (g) are the volume of MB solution and the mass of adsorbent sample, respectively.

### 2.5. Adsorption Experiments

The adsorption kinetic experiments were carried out in 100 mL flasks with the dispersion of 32.5 mg of the prepared samples in 50 mL of 200 mg·L^−1^ MB solution. Subsequently, these flasks were sealed using plastic wrap, put in the thermostatic shaker, and shaken for 0~120 min at 25 °C (160 rpm). Also, the adsorption isotherm experiments were performed in 100 mL flasks where 32.5 mg of the sample was dispersed in 50 mL, 0~300 mg·L^−1^ MB solution for 120 min. The concentration of MB and the adsorption quantities were determined using a UV/Vis spectrophotometer at λ = 664 nm and calculated following Equation (1). To estimate the regeneration properties, the MSH-1.75 sample after adsorption of MB was collected after being centrifugated, dried at 60 °C, and calcined at 600 °C for 4 h with a rate of 5 °C/min in air [22], then the same adsorption isotherm experiments were repeated by dispersing 32.5 mg of the regenerated samples in 50 mL of 100 mg·L^−1^ MB solution at 25 °C for 120 min.

All these experiments were carried out three times, and the average values were used to prepare the graphs in this work.

## 3. Results and Discussion

### 3.1. Characterization

**Figure 1:**
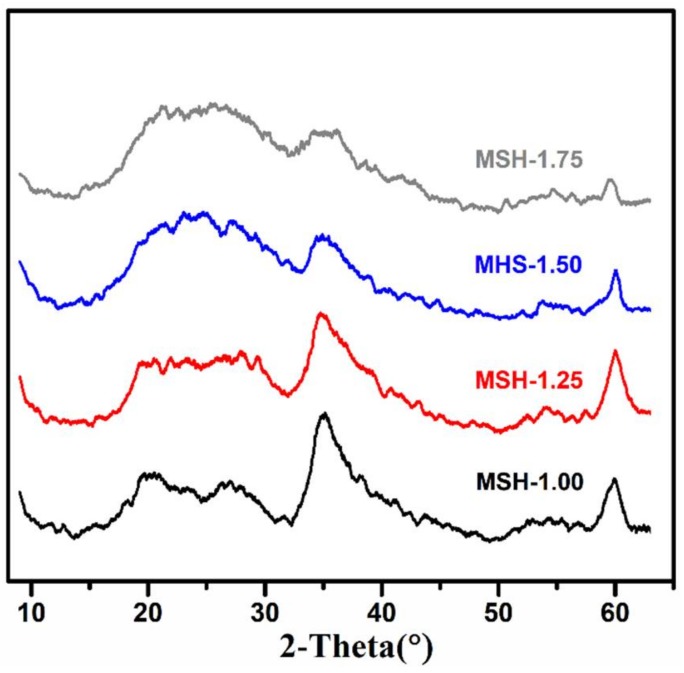
X-ray Diffraction (XRD) patterns of magnesium silicate hydrate samples (MSH) with different Si/Mg feeding ratios from 1.00 to 1.75.

Figure 1 shows the powder XRD patterns of four prepared MSH samples, which are very similar to those reported in the literature with three major broad peaks occurred at 2θ = 20°~30°, 35° and 60° for magnesium silicate hydrate (MSH) [23]. The low XRD diffraction densities result from the low crystallinity degree of the MSH as described in our previous work [7]. The chemical composition of four samples were further determined by ICP and EDX, and the corresponding results were summarized in Table 1 and Figure S1 (Supporting Information), respectively. One observes that all the four samples have similar chemical composition while the determined Si/Mg ratios slightly increase with the same trend as the Si/Mg feeding ratios, which are in the Si/Mg ratio range of the reported in the literature [24].

**Figure 2:**
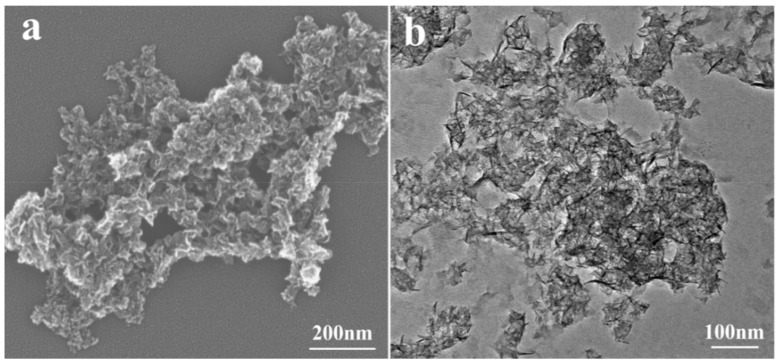
(**a**) Scanning electron microscope (SEM) and (**b**) Transmission electron microscope (TEM) images of MSH-1.75 sample.

Figure 2 and Figure S2 (Supporting Information) display the SEM and TEM images of four MSH samples. For all of four samples in Figure 2a and Figure S2, the morphologies from SEM are similar with the increase of Si/Mg feeding ratio from 1.00 to 1.75, and the samples present an irregular morphology with large particle size resulting from the aggregation. Figure 2b exhibits the TEM image of MSH-1.75, which are composed of lots of interlaced or aggregated nanosheets. Figure S3 (Supporting Information) further displays the particle size distributions of MSH samples tested by the laser particle size analyzer. The average particle size (d_50_) is 480, 530, 606, and 615 nm for MSH-1.00, MSH-1.25, MSH-1.50, and MSH-1.75, respectively. 

**Figure 3:**
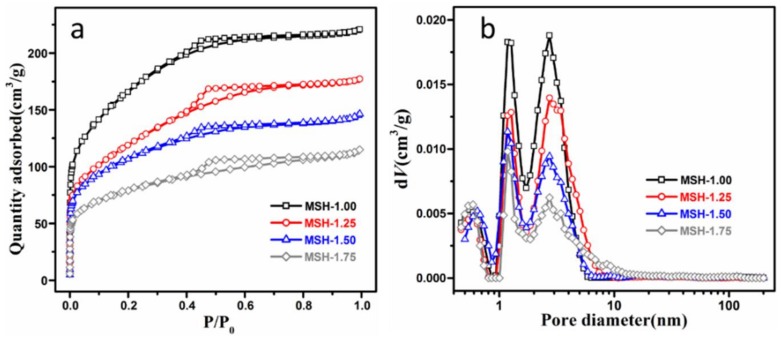
(**a**) N_2_ isothermal profiles of four MSH samples; (**b**) the pore size distribution of four samples calculated from the density functional theory (DFT).

Figure 3 illustrates the N_2_ adsorption/desorption isotherm and the pore-size distribution of four samples and all the Brunauer-Emmett-Teller (BET) data are listed in Table 1. In Figure 3a, all the samples exhibit the Type H2 hysteresis loop which is usually referred to pores like “ink bottle”. Besides, all the samples exhibit similar pore-size distribution in Figure 3b and Figure S4 (Supporting Information) and close average pore diameter in the range of 2.28–2.56 nm despite the difference in the Si/Mg feeding ratio. The surface area is decreased from 597 m^2^·g^−1^ for MSH-1.00 to 283 m^2^·g^−1^ for MSH-1.75.

### 3.2. Surface Charge and Adsorption Behavior

**Figure 4:**
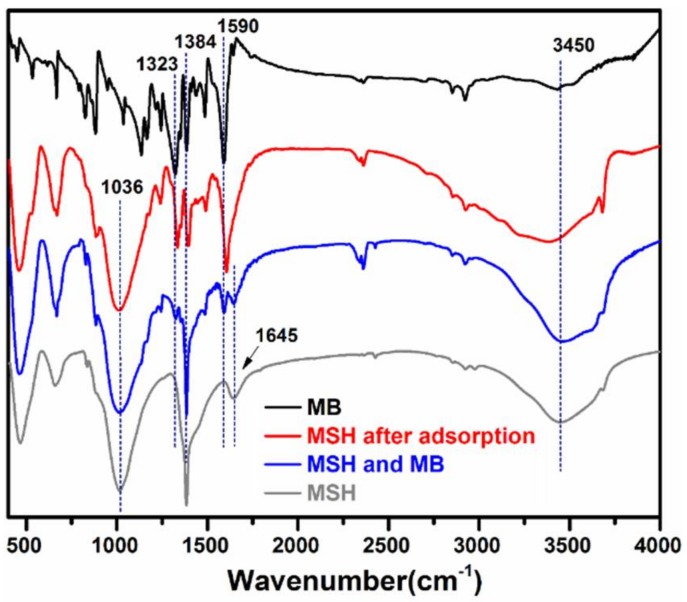
Fourier transform infrared spectroscopy (FT-IR) spectra of methylene blue (MB), mixture of MSH-1.75 and MB, and MSH-1.75 before and after adsorption of MB.

Figure 4 shows the FT-IR spectra of MB powder, mixture of MSH-1.75 and MB, and MSH-1.75 before and after adsorption of MB. Before the adsorption, MSH-1.75 exhibits some typical adsorption bands for MSH, for example, the adsorption bands at 464 and 1036 cm^−1^ are individually attributed to the Mg–O and Si–O stretching vibration while the bands at 1384, 1645 and 3450 cm^−1^ are due to the O–H vibrations [23]. After adsorption of MB, the characteristic absorption peak of aromatic rings at 1590 cm^−1^ and the C–N stretching vibrations at 1323 and 1384 cm^−1^ [25] shift to 1605, 1335 and 1395 cm^−1^, in comparison with MB. No similar change is observed for the spectrum of the mixture sample with MSH-1.75 and MB. These results confirm the existence of possible electrostatic interactions between MSH and MB after adsorption.

**Figure 5:**
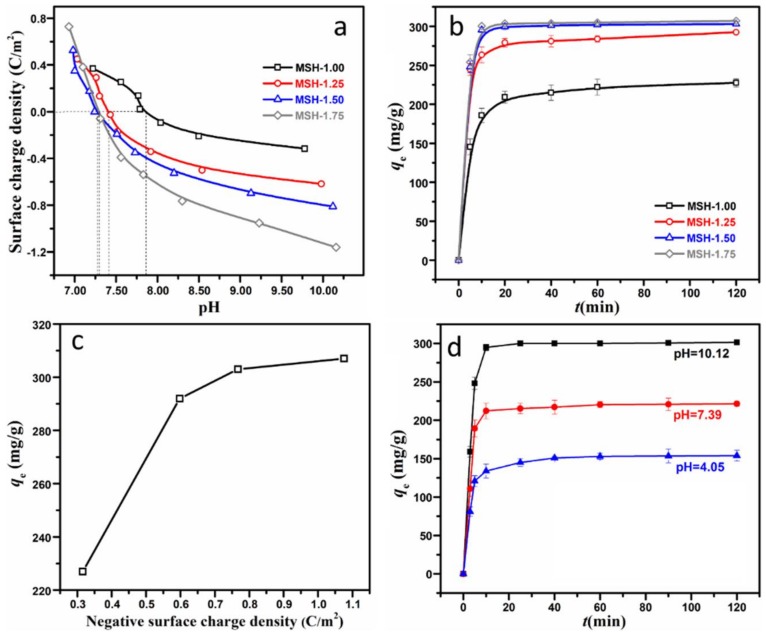
(**a**) Surface charge density of four MSH samples with different Si/Mg feeding ratio at different pH values; (**b**) equilibrium adsorption quantity of four MSH samples for MB; (**c**) the relationship between equilibrium adsorption quantity and surface charge density; (**d**) equilibrium adsorption quantity of MSH-1.75 sample at different pH values.

Generally, surface charge plays a key role during the adsorption process, especially for the electrostatic attraction mechanism. Figure 5a shows the potentiometric titration results for four MSH samples. Firstly, the pH value at the zero point of charge (pH_ZPC_) is 7.86, 7.41, 7.27 and 7.30 for MSH-1.00, -1.25, -1.50, and -1.75, respectively. When the pH value is higher than pH_ZPC_, all the MSH samples exhibit negative charge, resulting from the loss of the protons in the surface silanol groups [19], and furthermore the negative charge density is increased with the pH value. In comparison, moreover, the samples with higher Si/Mg ratio, such as MSH-1.75 and MSH-1.50, present higher negative surface charge density than MSH-1.25 and MSH-1.00 at the same pH value.

Figure 5b demonstrates the adsorption behavior of four MSH samples for MB. One observes that the adsorption of all the samples reaches the equilibrium in 120 min. However, there is a significant difference in the equilibrium adsorption quantity among these MSH samples, for example, as for MSH-1.50 and MSH-1.75 sample, more than 90% of the MB were removed in 10 min, and the equilibrium adsorption quantities at 120 min are 303 and 307 mg·g^−1^ respectively, which are much higher than 227 mg·g^−1^ for MSH-1.00. That is to say, the equilibrium adsorption quantity of MSH-1.75 is 0.35 times higher than that of MSH-1.00 although the surface area of MSH-1.00 (597 m^2^/g) is 1.1 times higher than MSH-1.75 (283 m^2^/g). Figure 5c further shows that the variation trend of the equilibrium adsorption quantities is the similar with that of the surface charge densities as described in Figure 5a. Therefore, here one may conclude that the surface charge is one of key parameters concerning the adsorption behavior of MSH for the MB. 

Figure 5d shows the difference in the adsorption quantity of MSH-1.75 at three different pH values under the same ion strength of the solution. The equilibrium adsorption quantity is 301 mg·g^−1^ at pH = 10.12, which is much higher than 221 mg·g^−1^ at pH = 7.39 and 154 mg·g^−1^ at pH = 4.05. As described in Figure 5a, the pH_ZPC_ of MSH-1.75 is 7.30, which is lower that pH = 10.12 and higher than pH = 4.05, and very close to the pH value of 7.39 in this experiment. That is to say, MSH-1.75 with negative surface charge density of −1.16 C·m^−2^ exhibits ca. 36% higher adsorption capacity related to the same sample at the zero point of charge. The results further provide another proof for the effect of the surface charge on the adsorption capacity.

**Figure 6:**
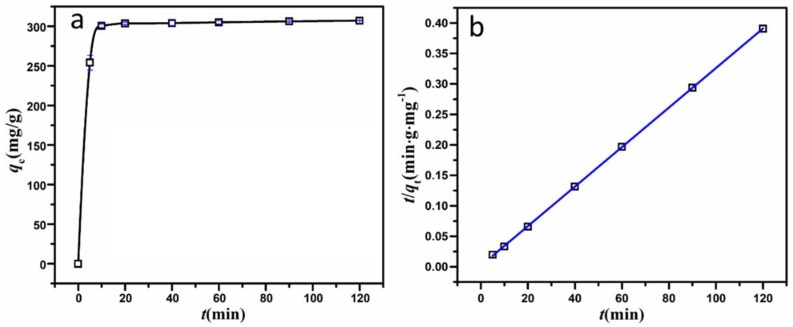
(**a**) Variation of adsorption quantity of MSH-1.75 as a function of contact time; and (**b**) the liner fitting of pseudo-second-order kinetics.

Figure 6 demonstrates the adsorption behavior of MSH-1.75 and the corresponding kinetic models for MB at *C*_0_ = 200 mg·L^−1^. As shown in Figure 6a, 98.7% of the MB in the solution are removed by MSH-1.75 in 10 min, and the adsorption process reaches the equilibrium in 20 min with the removal of 99.5% MB. Here, two typical adsorption kinetic models (the pseudo-first-order kinetic model shown as Equation (2) [26] and the pseudo-second-order kinetic model shown as Equation (3) [27]) were used to further analyze the adsorption mechanism of MSH-1.75 for MB. Also, the corresponding results are shown in Figure 6b and Figure S5 and summarized in Table 2.

(2) $$\mathrm{Pseudo-first-order}\;\mathrm{kinetic}:\;\log\left(q_{e}-q_{t}\right)=\log\left(q_{e}\right)-\frac{k_{1}}{2.303}t$$

(3) $$\mathrm{Pseudo-second-order}\;\mathrm{kinetic}:\;\frac{t}{q_{t}}=\frac{1}{k_{2}q_{e}^{2}}+\frac{t}{q_{e}}$$

where *q_e_* (mg·g^−1^) and *q_t_* (mg·g^−1^) are the adsorbed quantity for MB at equilibrium and at contact time *t* (min), respectively; *k*_1_ (min^−1^) and *k*_2_ (g·mg^−1^·min^−1^) is the rate constant for the pseudo-first-order kinetic model and the pseudo-first-order kinetic model. Through the linear relationship, the parameters such as *k*_1_, *k*_2_ and *q_e_* can be determined from the slope and intercept.

For MSH-1.75, the adsorption kinetic curves match the pseudo-second-order model well, with a better linear relationship in Figure 6b and a relatively higher *R*^2^ value, compared to the pseudo-first-order model in Figure S5 and Table 2. Also, the calculated *q*_e.cal_ values from the pseudo-second-order model are very close to the experimental data *q*_e,exp_.

**Figure 7:**
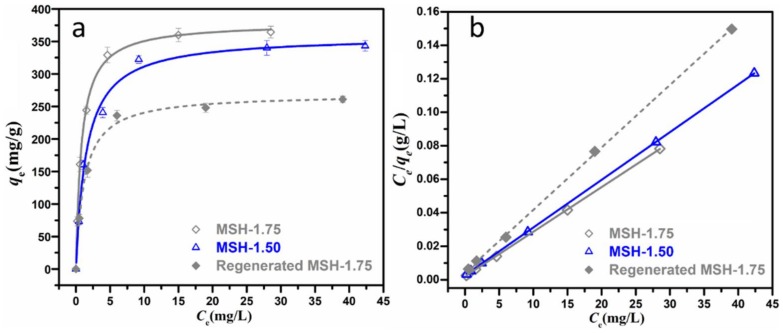
(**a**) Curve and (**b**) liner fitting of Langmuir isotherm for MB adsorption by MSH-1.50, MSH-1.75 and regenerated MSH-1.75 samples.

Figure 7 and Figure S6 show the adsorption isotherm of MSH-1.50 and MSH-1.75 for the MB in water after the adsorption for 120 min, and the corresponding results are expressed in the linear form of the Freundlich adsorption isotherm (Equation (4)) and Langmuir adsorption isotherm (Equation (5)) [28,29].

(4) $$\mathrm{Freundlich}:\;\log q_{e}=\log K_{F}+\frac{1}{n}\log C_{e}$$

(5) $$\mathrm{Langmuir}:\;\frac{C_{e}}{q_{e}}=\frac{1}{K_{L}q_{m}}+\frac{C_{e}}{q_{m}}$$

where *q_e_* (mg·g^−1^) and *q_m_* (mg·g^−1^) is equilibrium adsorption quantity and the maximum adsorption capacity, respectively; *C_e_* is the equilibrium concentration of MB in solution; *K_L_* is the equilibrium constant (L·mg^−1^); *K_F_* is the Freundlich isotherm constant and *n* presents the adsorption tendency. Through the linear relationship between *C_e_*/*q_e_* and *C_e_*, log*q_e_* and log*C_e_*, the other parameters: *q_m_*, *K_L_*, *K_F_* and *n*, can be calculated from the slope and intercept. 

One observes that the adsorption behavior of both MSH-1.50 and MSH-1.75 samples matches the Langmuir adsorption model much better than the Freundlich isotherm with the relatively higher *R*^2^ value as summarized in Table 3, suggesting that the adsorption of MB is much closer to the monolayer coverage on the surface of MSH sample [30]. The *q_m_* values of MSH-1.50 and MSH-1.75 based on the Langmuir adsorption model are 351 and 374 mg·g^−1^ respectively, and the *q_m_* value of 374 mg·g^−1^ for MSH-1.75 is the highest one among those reported in the literature as summarized in Table 4. Besides adsorption capacity, reusability is another crucial parameter for a high-performance absorbency [31]. Here, the regenerated MSH-1.75 sample exhibits a *q_m_* value of 268 mg·g^−1^ and remains ca. 72% of *q_m_*, suggesting promising reusability. Certainly, it still has large space to enhance reusability of the MSH by building a polymer network or porous substrate-supported composites for practical applications, although the reusability efficiency of 72% is higher than that of 60% for mesoporous magnesium silicate in ref [22]. The corresponding work is on the way. In brief, the high surface charge of the MSH sample significantly promotes the improvement of adsorption capacity for the removal of MB in aqueous solution. 

## 4. Conclusions

In this work, we successfully fabricated magnesium silicate hydrate (MSH) adsorbents with different surface charge by adjusting the Si/Mg feeding ratios, and examined the relationship between surface charge density and adsorption behavior for methylene blue (MB) in aqueous solution. The Si/Mg feeding ratios significantly modify the surface charge density of MSH, and then influence the adsorption capacity for MB in aqueous solution. Among the investigated four samples, MSH-1.75 has the highest negative surface charge density of −1.16 C·m^−2^ and exhibits ca. 36% higher adsorption capacity related to the same sample at the zero point of charge. With the largest adsorption capacity of 374 mg·g^−1^ calculated from the Langmuir isotherm, furthermore, the MSH materials show good regeneration ability with ca. 72% of reusability efficiency. Therefore, this is an available route to improve the removal capacity of magnesium silicate hydrate for cationic dyes by increasing the corresponding negative surface charge density. Furthermore, magnesium silicate hydrate is one kind of promising material for removing dye pollution from wastewater.

## Figures and Tables

**Table 1:** The inductively coupled plasma (ICP), energy-dispersive X-ray spectroscopy (EDX), and pore structure analysi results of four samples with different feeding Si/Mg ratio.

Samples	Si/Mg Molar Ratio Tested by ICP	Si/Mg Molar Ratio Tested by EDX	Surface Area (m^2^·g^−1^)	Average Pore Diameter (nm)
MS-1.00	1.06	1.09	597	2.28
MS-1.25	1.20	1.23	428	2.56
MS-1.50	1.41	1.43	381	2.37
MS-1.75	1.66	1.73	283	2.51

**Table 2:** Pseudo-first-order and pseudo-second-order adsorption kinetic constants for MB adsorption.

Sample	*q_e_*_,exp_ (mg·g^−1^)	Pseudo-First-Order Model			Pseudo-Second-Order Model		
		*q_e_*_,cal_ (mg·g^−1^)	*k*_1_	*R* ^2^	*q_e_*_,cal_ (mg·g^−1^)	*k*_2_	*R* ^2^
MSH-1.75	307	50.3	0.941	0.786	308	0.00552	0.999

**Table 3:** Langmuir and Freundlich isotherms parameters of MSH-1.50, MSH-1.75 and regenerated MSH-1.75 samples for MB adsorption.

Sample	Langmuir			Freundlich		
	*q_m_*	*K_L_*	*R* ^2^	*n*	*K_F_*	*R* ^2^
MSH-1.50	351	1.04	0.999	3.59	146	0.834
MSH-1.75	374	1.34	0.999	3.31	166	0.820
Regenerated MSH-1.75	268	0.84	0.999	-	-	-

**Table 4:** Summaries of adsorption capacity of various adsorbents for MB under a certain condition.

Adsorbents	Equilibrium Time	pH Value	Surface Area (m^2^·g^−1^)	Adsorption Capacity (mg·g^−1^)	Ref.
MgSi hollow spheres	30 min	-	521	207	[32]
Florisil	4 h	-	250	149.3	[17]
t-yolk-shell magnetic MgSi	15 h	-	293	188	[11]
MgSi nanotubes	-	-	649	276	[4]
Diatomite	48 h	11	27.8	198	[19]
Activated carbon	90 min	-	984	68.72	[33]
Graphene-c-MWCNT hybrid aerogel	-	-	435	190.0	[34]
Copper silicate hollow nanotubes	-	-	518	173	[35]
Modified montmorillonite clays	-	-	-	71	[21]
GO-CS hydrogels	58 h	6.5	-	350	[36]
MSH-1.75	2 h	10.1	283	374	this work

## Supplementary Materials

The following are available online at http://www.mdpi.com/2079-4991/8/5/271/s1.

## References

[1] Rane N.R., Chandanshive V.V., Khandare R.V., Gholave A.R., Yadav S.R., Govindwar S.P. (2014). Green Remediation of Textile Dyes Containing Wastewater by *Ipomoea hederifolia* L.. RSC Adv..

[2] Extremera R., Pavlovic I., Pérez M.R., Barriga C. (2012). Removal of Acid Orange 10 by Calcined Mg/Al Layered Double Hydroxides from Water and Recovery of the Adsorbed Dye. Chem. Eng. J..

[3] Razali M., Kim J.F., Attfield M., Budd P.M., Drioli E., Lee Y.M., Szekely G. (2015). Sustainable Wastewater Treatment and Recycling in Membrane Manufacturing. Green Chem..

[4] Qu J., Li W., Cao C.-Y., Yin X.-J., Zhao L., Bai J., Qin Z., Song W.-G. (2012). Metal Silicate Nanotubes with Nanostructured Walls as Superb Adsorbents for Uranyl Ions and Lead Ions in Water. J. Mater. Chem..

[5] Özcan A., Öncü E.M., Özcan A.S. (2006). Kinetics, Isotherm and Thermodynamic Studies of Adsorption of Acid Blue 193 from Aqueous Solutions onto Natural Sepiolite. Colloids Surf. A.

[6] Lu Q., Li Q., Zhang J., Li J., Lu J. (2016). Facile Mesoporous Template-Assisted Hydrothermal Synthesis of Ordered Mesoporous Magnesium Silicate as an Efficient Adsorbent. Appl. Surf. Sci..

[7] Huang R., Wu M., Zhang T., Li D., Tang P., Feng Y. (2017). Template-Free Synthesis of Large-Pore-Size Porous Magnesium Silicate Hierarchical Nanostructures for High-Efficiency Removal of Heavy Metal Ions. ACS Sustain. Chem. Eng..

[8] Narasimharao K., Ali T.T., Bawaked S., Basahel S. (2014). Effect of Si Precursor on Structural and Catalytic Properties of Nanosize Magnesium Silicates. Appl. Catal. A Gen..

[9] Gui C.X., Wang Q.Q., Hao S.M., Qu J., Huang P.P., Cao C.Y., Song W.G., Yu Z.Z. (2014). Sandwichlike Magnesium Silicate/Reduced Graphene Oxide Nanocomposite for Enhanced Pb^2+^ and Methylene Blue Adsorption. ACS Appl. Mater. Interfaces.

[10] Cao C.Y., Wei F., Qu J., Song W.G. (2013). Programmed Synthesis of Magnetic Magnesium Silicate Nanotubes with High Adsorption Capacities for Lead and Cadmium Ions. Chem. Eur. J..

[11] Zheng J., Cheng C., Yan R.-W., Fang W.-J., Chen C., Huai H.-X., Wang C.-C. (2014). Synthesis of Yolk–Shell Magnetic Magnesium Silicate with Tunable Yolk Morphology for Removal of Methylene Blue in Water. J. Alloys Compd..

[12] Yari S., Abbasizadeh S., Mousavi S.E., Moghaddam M.S., Moghaddam A.Z. (2015). Adsorption of Pb(II) and Cu(II) Ions from Aqueous Solution by an Electrospun CeO_2_ Nanofiber Adsorbent Functionalized with Mercapto Groups. Process Saf. Environ..

[13] Tripathi S., Bose R., Roy A., Nair S., Ravishankar N. (2015). Synthesis of Hollow Nanotubes of Zn_2_SiO_4_ or SiO_2_: Mechanistic Understanding and Uranium Adsorption Behavior. ACS Appl. Mater. Interfaces.

[14] Homaeigohar S., Zillohu A.U., Abdelaziz R., Hedayati M.K., Elbahri M. (2016). A Novel Nanohybrid Nanofibrous Adsorbent for Water Purification from Dye Pollutants. Materials.

[15] Janaki V., Oh B.-T., Shanthi K., Lee K.-J., Ramasamy A.K., Kamala-Kannan S. (2012). Polyaniline/Chitosan Composite: An Eco-Friendly Polymer for Enhanced Removal of Dyes from Aqueous Solution. Synth. Met..

[16] Dogan M., Alkan M., Turkyilmaz A., Ozdemir Y. (2004). Kinetics and Mechanism of Removal of Methylene Blue by Adsorption onto Perlite. J. Hazard. Mater..

[17] Ferrero F. (2010). Adsorption of Methylene Blue on Magnesium Silicate: Kinetics, Equilibria and Comparison with Other Adsorbents. J. Environ. Sci..

[18] Ravikovitch P.I., Haller G.L., Neimark A.V. (1998). Density Functional Theory Model for Calculating Pore Size Distributions: Pore Structure of Nanoporous Catalysts. Adv. Colloid Interface Sci..

[19] Al-Ghouti M.A., Khraisheh M.A.M., Allen S.J., Ahmad M.N. (2003). The Removal of Dyes from Textile Wastewater: A Study of the Physical Characteristics and Adsorption Mechanisms of Diatomaceous Earth. J. Environ. Manag..

[20] Wang W., Tian G., Zhang Z., Wang A. (2015). A Simple Hydrothermal Approach to Modify Palygorskite for High-Efficient Adsorption of Methylene Blue and Cu(II) Ions. Chem. Eng. J..

[21] Cottet L., Almeida C.A.P., Naidek N., Viante M.F., Lopes M.C., Debacher N.A. (2014). Adsorption Characteristics of Montmorillonite Clay Modified with Iron Oxide with Respect to Methylene Blue in Aqueous Media. Appl. Clay Sci..

[22] Zhang J., Dang L., Zhang M., Lu Q., Zhao S. (2017). Characterization of Mesoporous Magnesium Silicate with Hierarchical Structure and Its Adsorption Performance for Dye and Lead Ion. Surf. Interfaces.

[23] Brew D.R.M., Glasser F.P. (2005). Synthesis and Characterisation of Magnesium Silicate Hydrate Gels. Cem. Concr. Res..

[24] Bernard E., Lothenbach B., Rentsch D., Pochard I., Dauzères A. (2017). Formation of Magnesium Silicate Hydrates (M-S-H). Phys. Chem. Earth.

[25] Wu Z., Zhong H., Yuan X., Wang H., Wang L., Chen X., Zeng G., Wu Y. (2014). Adsorptive Removal of Methylene Blue by Rhamnolipid-Functionalized Graphene Oxide from Wastewater. Water Res..

[26] Zhang M., Song L., Jiang H., Li S., Shao Y., Yang J., Li J. (2017). Biomass Based Hydrogel as An Adsorbent for the Fast Removal of Heavy Metal Ions from Aqueous Solutions. J. Mater. Chem. A.

[27] Yu R., Shi Y., Yang D., Liu Y., Qu J., Yu Z.Z. (2017). Graphene Oxide/Chitosan Aerogel Microspheres with Honeycomb-Cobweb and Radially Oriented Microchannel Structures for Broad-Spectrum and Rapid Adsorption of Water Contaminants. ACS Appl. Mater. Interfaces.

[28] Chen Y., He M., Wang C., Wei Y. (2014). A Novel Polyvinyltetrazole-Grafted Resin with High Capacity for Adsorption of Pb(II), Cu(II) and Cr(III) Ions from Aqueous Solutions. J. Mater. Chem. A.

[29] Ghaemi A., Torab-Mostaedi M., Ghannadi-Maragheh M. (2011). Characterizations of Strontium(II) and Barium(II) Adsorption from Aqueous Solutions Using Dolomite Powder. J. Hazard. Mater..

[30] Pan Y., Liu Z., Wang W., Peng C., Shi K., Ji X. (2016). Highly Efficient Macroporous Adsorbents for Toxic Metal Ions in Water Systems Based on Polyvinyl Alcohol–Formaldehyde Sponges. J. Mater. Chem. A.

[31] Kupai J., Razali M., Buyuktiryaki S., Kecili R., Szekely G. (2017). Long-Term Stability and Reusability of Molecularly Imprinted Polymers. Polym. Chem..

[32] Wang Y., Wang G., Wang H., Liang C., Cai W., Zhang L. (2010). Chemical-Template Synthesis of Micro/Nanoscale Magnesium Silicate Hollow Spheres for Waste-Water Treatment. Chem. Eur. J..

[33] Kumar P.S., Ramalingam S., Sathishkumar K. (2010). Removal of Methylene Blue Dye from Aqueous Solution by Activated Carbon Prepared from Cashew Nut Shell as a New Low-Cost Adsorbent. Korean J. Chem. Eng..

[34] Sui Z., Meng Q., Zhang X., Ma R., Cao B. (2012). Green Synthesis of Carbon Nanotube–Graphene Hybrid Aerogels and Their Use as Versatile Agents for Water Purification. J. Mater. Chem..

[35] Zhang M., Wang B., Zhang Y., Li W., Gan W., Xu J. (2016). Facile Synthesis of Magnetic Hierarchical Copper Silicate Hollow Nanotubes for Efficient Adsorption and Removal of Hemoglobin. Dalton Trans..

[36] Chen Y., Chen L., Bai H., Li L. (2013). Graphene Oxide–Chitosan Composite Hydrogels as Broad-Spectrum Adsorbents for Water Purification. J. Mater. Chem. A.

