# Supplementary material for: Fabrication and Adsorption Behavior of Magnesium Silicate Hydrate Nanoparticles towards Methylene Blue
Source: Nanomaterials (Basel). 2018 Apr 24;8(5):271. doi: 10.3390/nano8050271 (PMC5977285; doi:10.3390/nano8050271)
Supplement: Supplementary file 1 [file nanomaterials-08-00271-s001.pdf]

Supporting information

Fabrication and Adsorption Behavior of Magnesium Silicate Hydrate Nanoparticles towards Methylene Blue

Renyao Huang [1], Li He [1], Tao Zhang [2], Dianqing Li [1], Pinggui Tang [1], Yingying Zhao [1,*] and Yongjun Feng [1,*]

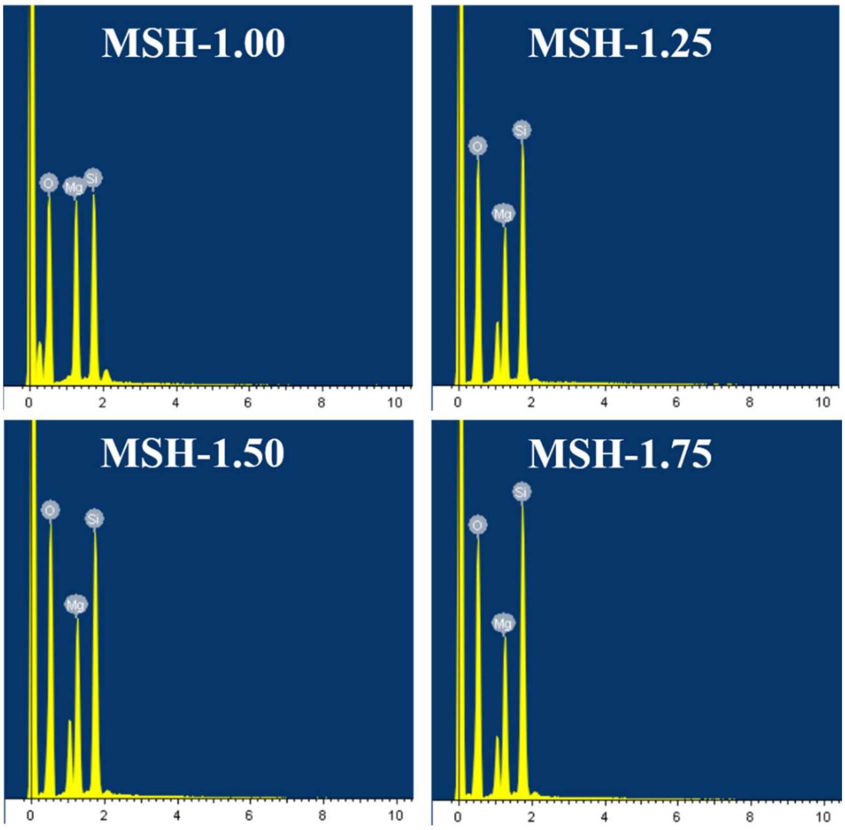

Figure S1. EDX of four magnesium silicate hydrate samples from MSH-1.00 to -1.75.

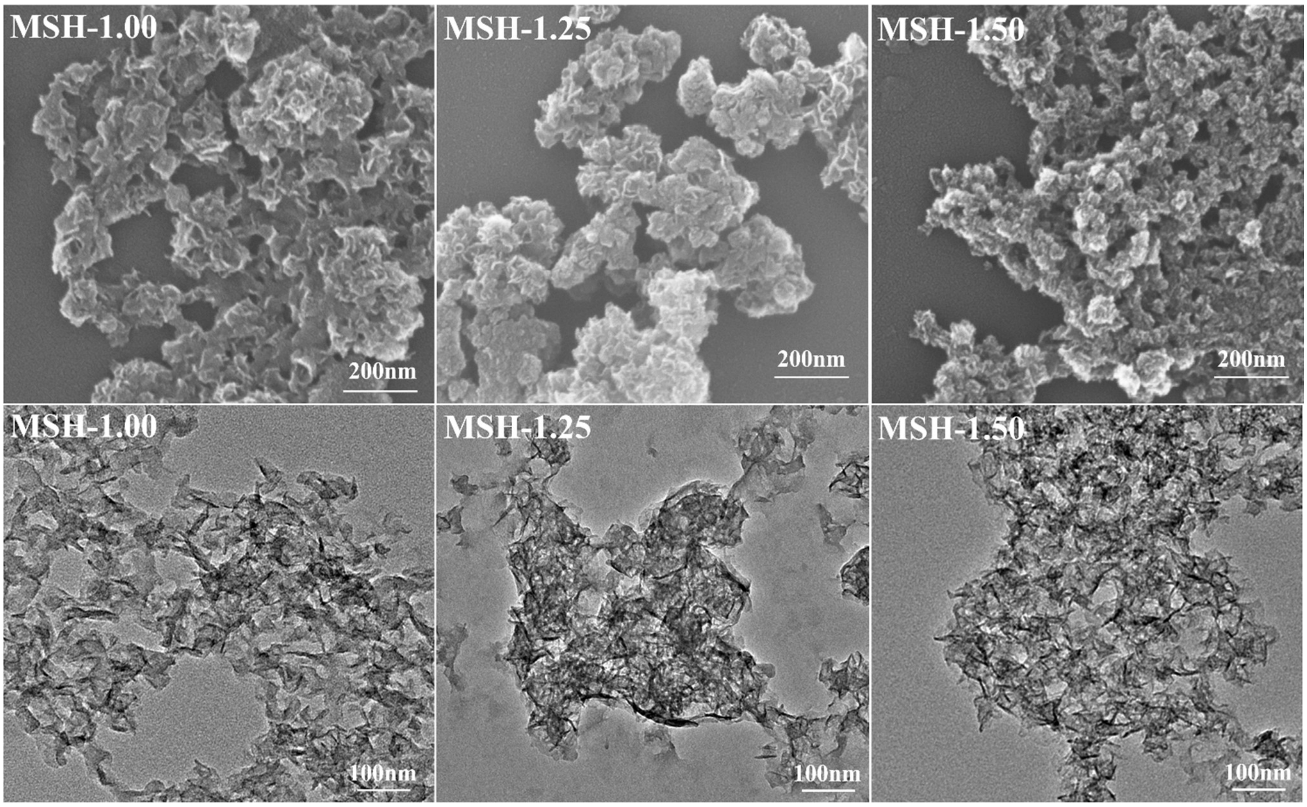

Figure S2. SEM images of three MSH samples with different Si/Mg ratios from 1.00 to 1.50.

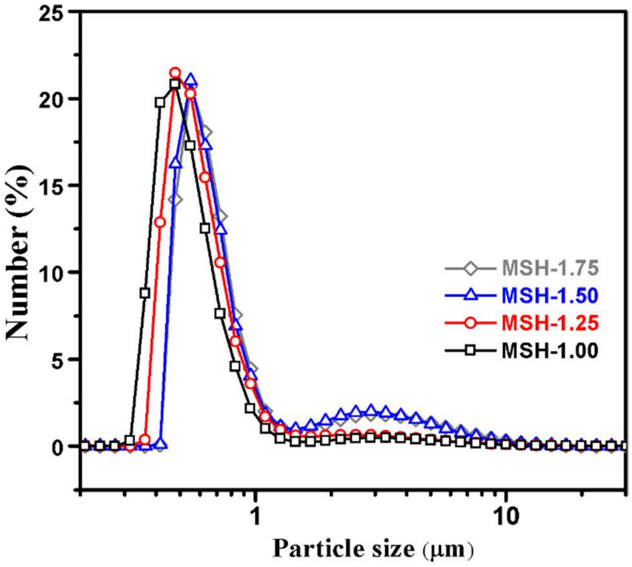

Figure S3. Particle size distribution of four magnesium silicate hydrate samples from MSH-1.00 to MSH-1.75.

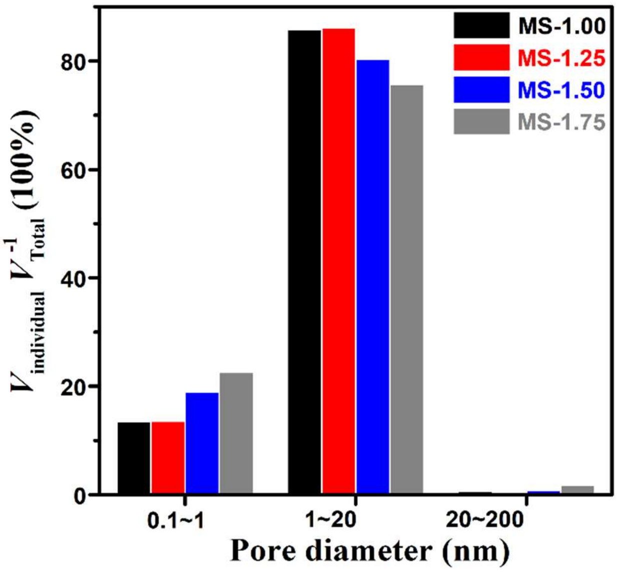

Figure S4. The ratio of pore volume in a certain range of pore size over total pore volume as a function of pore diameter.

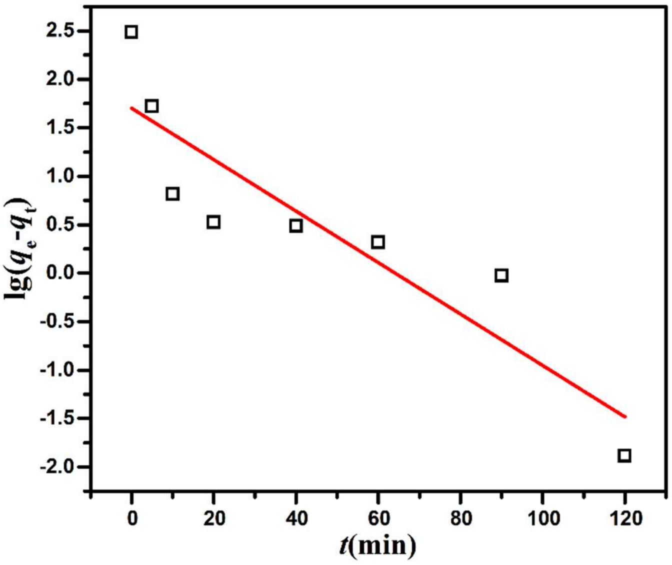

Figure S5. The liner fitting of pseudo-first-order kinetics of MSH-1.75 for MB adsorption.

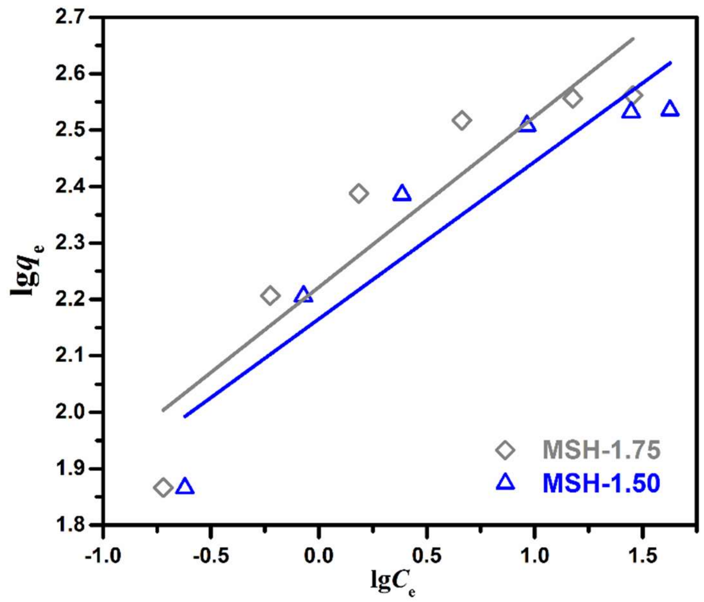

Figure S6. The liner fitting of Freundlich isotherm for MB adsorption by MSH-1.50 and MSH-1.75.
